# Pericardial late gadolinium enhancement and time to recurrence: a substudy from RHAPSODY, a phase 3 clinical trial of rilonacept in recurrent pericarditis

**DOI:** 10.1093/ehjimp/qyad003

**Published:** 2023-05-26

**Authors:** Paul C Cremer, David Lin, Sushil A Luis, John Petersen, Antonio Abbate, Christine L Jellis, Debbie Kwon, Antonio Brucato, Fang Fang, Antonella Insalaco, Martin LeWinter, Basil S Lewis, Liangxing Zou, Stephen J Nicholls, Allan L Klein, Massimo Imazio, John F Paolini, RHAPSODY Investigators, Antonio Abbate, Wael Abo-Auda, Asif Akhtar, Michael Arad, Shaul Atar, Bipul Baibhav, Karan Bhalla, Antonio Brucato, Sean Collins, David Colquhoun, Paul Cremer, David Cross, Girish Dwivedi, Alon Eisen, Nahum Freedberg, Shmuel Fuchs, Eliyazar Gaddam, Marco Gattorno, Eli Gelfand, Paul Grena, Majdi Halabi, David Harris, Massimo Imazio, Antonella Insalaco, Amin Karim, Allan Klein, Kirk Knowlton, Apostolos Kontzias, Robert Kornberg, Faisal Latif, David Leibowitz, Martin LeWinter, David Lin, Dor Lotan, Pey Wen Lou, S. Allen Luis, Mady Moriel, Stephen Nicholls, John Petersen, Michael Portman, Philip Roberts-Thomson, Elad Schiff, Robert Siegel, Michael Stokes, Paul Sutej, Samuel Wittekind, Valentin Witzling, Robert Zukermann

**Affiliations:** Center for the Diagnosis and Treatment of Pericardial Diseases, Section of Cardiovascular Imaging, Department of Cardiovascular Medicine, Heart, Vascular, and Thoracic Institute, Cleveland Clinic, 9500 Euclid Avenue, Desk J1-5, Cleveland, OH 44195, USA; Minneapolis Heart Institute, Abbott Northwestern Hospital, Minneapolis, MN, USA; Division of Cardiovascular Ultrasound, Department of Cardiovascular Medicine, Mayo Clinic, Rochester, MN, USA; Swedish Medical Center, Seattle, WA, USA; Pauley Heart Center, Virginia Commonwealth University, Richmond, VA, USA; Center for the Diagnosis and Treatment of Pericardial Diseases, Section of Cardiovascular Imaging, Department of Cardiovascular Medicine, Heart, Vascular, and Thoracic Institute, Cleveland Clinic, 9500 Euclid Avenue, Desk J1-5, Cleveland, OH 44195, USA; Center for the Diagnosis and Treatment of Pericardial Diseases, Section of Cardiovascular Imaging, Department of Cardiovascular Medicine, Heart, Vascular, and Thoracic Institute, Cleveland Clinic, 9500 Euclid Avenue, Desk J1-5, Cleveland, OH 44195, USA; Department of Biomedical and Clinical Science, University of Milan, Fatebenefratelli Hospital, Milan, Italy; Kiniksa Pharmaceuticals Corp., Lexington, MA, USA (at time of study); Division of Rheumatology, IRCCS Ospedale Pediatrico Bambino Gesù, Rome, Italy; Cardiology Unit, University of Vermont Medical Center, Burlington, VT, USA; Cardiovascular Clinical Research Institute, Lady Davis Carmel Medical Center and Technion–Israel Institute of Technology, Haifa, Israel; Kiniksa Pharmaceuticals Corp., Lexington, MA, USA (at time of study); Monash Cardiovascular Research Centre, Victorian Heart Institute, Monash University, Clayton, Victoria, Australia; Center for the Diagnosis and Treatment of Pericardial Diseases, Section of Cardiovascular Imaging, Department of Cardiovascular Medicine, Heart, Vascular, and Thoracic Institute, Cleveland Clinic, 9500 Euclid Avenue, Desk J1-5, Cleveland, OH 44195, USA; Cardiothoracic Department, University Hospital ‘Santa Maria della Misericordia,’ ASUFC, Udine, Italy; Kiniksa Pharmaceuticals Corp., Lexington, MA, USA (at time of study)

**Keywords:** rilonacept, pericarditis, recurrence, cardiac MRI

## Abstract

**Aims:**

In this protocol-predefined substudy of the RHAPSODY trial, the primary aim was to assess whether pericardial late gadolinium enhancement (LGE) was associated with time to pericarditis recurrence.

**Methods and results:**

RHAPSODY was a Phase 3 double-blind, placebo-controlled, randomized-withdrawal trial that demonstrated the efficacy of rilonacept in recurrent pericarditis (RP). Patients with a history of multiple RP and an active recurrence were enrolled and had the option to participate in a cardiac magnetic resonance (CMR) imaging substudy. CMRs were interpreted by a blinded independent core laboratory with prespecified criteria to define pericardial LGE. Compared to patients with trace or mild pericardial LGE (*n* = 9), patients with moderate or severe pericardial LGE (*n* = 16) generally had a higher number of recurrent episodes per year (5.3 vs. 3.9) and a higher mean CRP level (3.6 vs. 1.1 mg/dL). Overall, 10/14 (71.4%) who received a placebo had a recurrence compared to 0/11 (0%) who received rilonacept. In patients randomized to placebo who had moderate or severe pericardial LGE, the median time to recurrence was 4.2 weeks compared to 10.7 weeks in patients who had trace or mild pericardial LGE. At the conclusion of the event-driven randomized-withdrawal period, among patients receiving a placebo, 5/7 (71.4%) with trace or mild pericardial LGE and 5/7 (71.4%) with moderate or severe pericardial LGE had a recurrence.

**Conclusions:**

Among patients with multiple RP, these preliminary findings support the concept of pericardial LGE as an imaging biomarker that may inform the duration of treatment and risk of recurrence with cessation of therapy and larger studies should be considered.

**ClinicalTrials.gov Identifier:**

NCT03737110

## Lay Summary

Patients with recurrent pericarditis (RP) can suffer from debilitating pain and a poor quality of life. Rilonacept blocks interleukin 1 (IL-1), the major inflammatory driver of RP, and is highly effective at treating active episodes of RP and preventing recurrence. In pericarditis, there is the recruitment of blood vessels to the pericardium, and the extent of these new blood vessels tracks with the degree of inflammation. Cardiac magnetic resonance imaging (CMR) readily images this blood supply and can therefore assess inflammation by the magnitude of pericardial late gadolinium enhancement (LGE). In this study of RP patients with CMR, no patients who continued rilonacept had a recurrence compared to 10/14 (71.4%) patients who stopped rilonacept and received a placebo. In the patients who received a placebo, the rate of eventual recurrence was similar among patients with trace or mild pericardial LGE at baseline (5/7) compared to patients with moderate or severe pericardial LGE at baseline (5/7). However, patients who demonstrated moderate or severe pericardial LGE had a faster recurrence (∼4 weeks after stopping rilonacept) compared to patients with trace or mild pericardial LGE (∼11 weeks after stopping rilonacept). These results suggest that pericardial LGE can serve as an imaging biomarker to assess the severity of RP and raise the possibility that CMR could be studied in future clinical trials to determine appropriate therapy and treatment duration in patients with RP.

## Graphical Abstract

**Graphical abstract:**
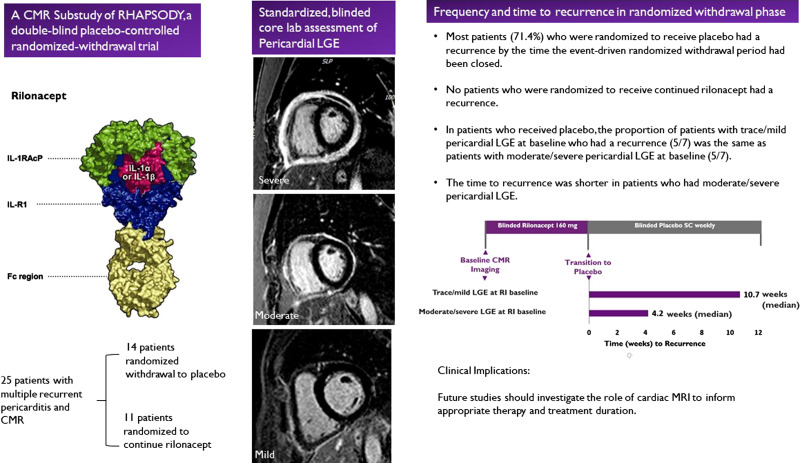

Recurrent pericarditis (RP) is a debilitating auto-inflammatory disease associated with substantial morbidity and typically requires prolonged treatment.^1^ Interleukin 1 (IL-1) is a cytokine that has been implicated as a key mediator of RP. RHAPSODY (NCT03737110) was a Phase 3, international, multicenter, double-blind, placebo-controlled, randomized-withdrawal trial which demonstrated that once-weekly rilonacept, an IL-1α and IL-1β cytokine trap, effectively treated RP episodes and reduced risk of recurrence.^2^ The protocol was approved by relevant institutional review boards or independent ethics committees for all sites, and all patients provided written informed consent.

In patients with RP, CMR imaging may inform the disease course, as late gadolinium enhancement (LGE) of the pericardium indicates neovascularization and is associated with acute or persistent inflammation.^3^ Accordingly, we performed a protocol-predefined hypothesis-generating substudy in RHAPSODY to assess whether the magnitude of baseline pericardial LGE was related to the frequency of or time to recurrence.

In RHAPSODY, patients with a history of multiple RP episodes presenting with an acute recurrence episode despite standard therapy were initiated on rilonacept treatment during a 12-week run-in (RI) period, and background medications were discontinued.^2^ Optional baseline pericardial CMR was performed at select centers within 7 days before rilonacept initiation. According to a standard protocol, LGE images were obtained in long- and short-axis orientations ∼10–20 min after intravenous injection of gadolinium-based contrast agent using a phase-sensitive inversion recovery (PSIR) technique with an inversion time selected for optimal nulling of the myocardium. CMR images were reviewed by two blinded imaging cardiologists (D.K. and C.L.J.) with level III expertise in accordance with the Society of Cardiovascular Magnetic Resonance. Pericardial LGE was graded using previously described criteria^4^: none/trace [if present, increased pericardial signal limited to ≤50% of the cardiac circumference at 1 of 3 left ventricle levels (base/mid-cavity/apex)], mild (increased pericardial signal involving >50% of the cardiac circumference at 1 of 3 ventricle levels), moderate (increased pericardial signal involving >50% of the cardiac circumference at 2 of three ventricle levels), or severe (increased pericardial signal involving >50% of the cardiac circumference at all three ventricle levels). The following CMR scanners were used: Philips Achieva 1.5T, Philips Ingenia 3T, Siemens Aera 1.5T, Siemens Magnetom Vida 3T, GE Optima MR450w 1.5T, and GE Signa HDxt 1.5T. CMR analysis was performed using CVI42 (Circle Cardiovascular Imaging Inc. Calgary, Canada). All studies were re-analyzed in a blinded fashion with good intra-observer {Kappa statistics: 0.93 and 0.77; intraclass correlation coefficients (ICC): 0.92 [95% Confidence Interval (CI) 0.79–0.97] and 0.79 (95% CI 0.48–0.92)} and inter-observer [Kendall’s coefficient of concordance, 0.88; ICC, 0.88 (95% CI 0.61–0.95)] reproducibility. To assess the risk for recurrence, univariable Cox proportional hazards were performed to compare patients with moderate or severe pericardial LGE to patients with trace or mild pericardial LGE. All data are included in the submission/manuscript file. Statistical analyses were performed with SAS version 9.4 and R Core Team (2019).

Patients meeting prespecified response criteria at the end of the RI period were randomized to further treatment with rilonacept or placebo. An RP event was defined as a return of pericarditis pain with an increase in C-reactive protein (CRP) level, as well as supportive objective evidence (e.g. pericardial effusion, pericardial rub, or electrocardiographic changes); each event was confirmed by an independent clinical-events committee. The number and timing of RP events within treatment groups were compared descriptively according to baseline pericardial LGE severity.

Twenty-five of 86 randomized patients had undergone CMR at RI baseline (subsequent randomization: 14 placebo, 11 rilonacept), all while experiencing an acute pericarditis recurrence. Patients with moderate/severe pericardial LGE (rilonacept, *n* = 9; placebo, *n* = 7) generally had a longer mean duration of RP (2.8 vs. 1.9 years), a higher mean number of recurrent episodes per year (5.3 vs. 3.9), and higher mean CRP level at baseline (3.6 vs. 1.1 mg/dL), compared with patients having trace/mild pericardial LGE (rilonacept, *n* = 2; placebo, *n* = 7). Only three patients had significant pericardial effusions, defined as greater than trace/physiologic. One patient had a moderate pericardial effusion (1.0–2.0 cm), one patient had a large pericardial effusion (2.0–2.5 cm), and one patient had a very large pericardial effusion (>2.5 cm). All of these patients had severe pericardial LGE. Among the 25 patients, RP occurred in 10/14 (71.4%) placebo-randomized patients and 0/11 rilonacept-randomized patients, demonstrating the efficacy of rilonacept. Among placebo patients, the median time to recurrence was 4.2 weeks in those with moderate/severe pericardial LGE at baseline vs. 10.7 weeks in those with trace/mild pericardial LGE at baseline, highlighting the incremental prognostic value of baseline CMR (*Figure 1*). The univariable Cox proportional hazards ratio for recurrence among patients treated with placebo who had moderate or severe pericardial LGE was 2.50 (95% CI 0.64–9.85) (*P* = 0.17). However, by the closure of the event-driven randomized-withdrawal period (triggered by the accrual of a prespecified number of adjudicated first post-randomization recurrence events),^2^ the proportion of placebo-randomized patients with recurrence was the same regardless of trace/mild or moderate/severe pericardial LGE at baseline (5/7, 71.4% in both).

**Figure 1:**
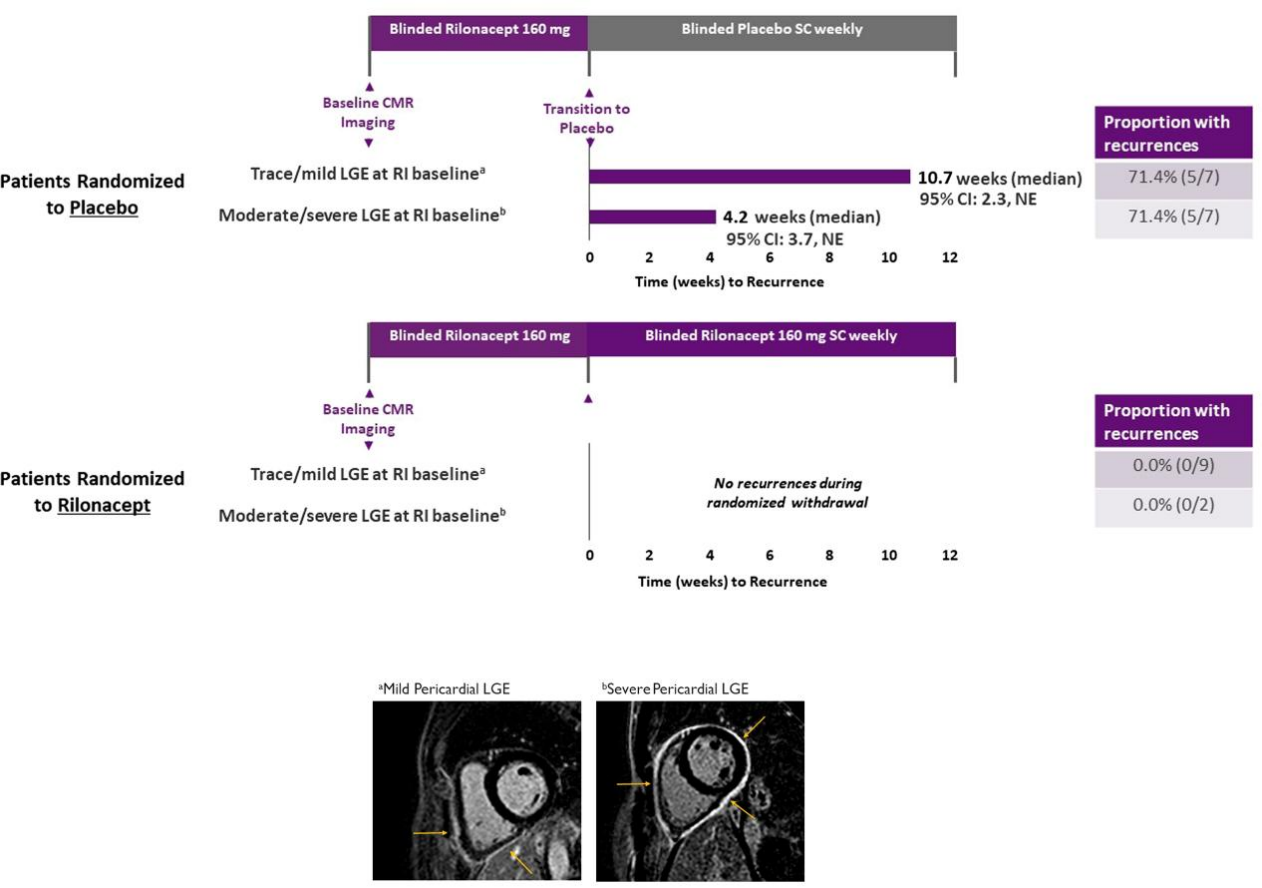
Time to pericarditis recurrence by treatment and baseline pericardial LGE severity. Time to pericarditis recurrence was defined as time from randomization to placebo or rilonacept (following a 12-week RI of rilonacept treatment in all patients) to date of first recurrence. Accompanying footnoted images are illustrative CMR-PSIR short-axis sequences from two representative cases with mild^a^ or severe^b^ pericardial LGE at baseline (arrows indicate pericardial LGE). CI, confidence interval; CMR, cardiac magnetic resonance imaging; LGE, late gadolinium enhancement; NE, not estimable; PSIR, phase-sensitive inversion recovery; RI, run-in; SC, subcutaneous.

The major limitation of this hypothesis-generating CMR substudy of RHAPSODY was the small sample size and few events. The wide confidence intervals related to the primary outcome of risk of recurrence reflect that this study was underpowered. A notable strength is that CMR interpretation was performed independently and blinded to clinical data within the context of an international trial with standardized assessments and outcomes. Among patients who washed off of rilonacept through randomization to placebo, time to pericarditis recurrence was numerically shorter for patients with moderate/severe vs. trace/mild pericardial LGE on acute presentation (baseline).

Prior retrospective cohort studies have demonstrated the potential prognostic value of pericardial LGE in pericarditis, in particular by providing a quantitative framework,^5^ an approach that would require standardization across laboratories for broader clinical application. Our findings generally support the concept of pericardial LGE as a potential imaging biomarker that may inform the duration of treatment in RP by identifying the propensity for recurrence upon withdrawal of therapy in an otherwise clinically stable patient. Moreover, the qualitative methodology used for grading pericardial LGE tracked with disease severity and is straightforward and reproducible. Further study of this methodology in a larger patient cohort and with adjustment for clinical variables is warranted.

## Lead author biography

Paul C. Cremer MD is a cardiologist and cardiovascular imager at the Cleveland Clinic where he is the associate director of the cardiac intensive care unit and the cardiovascular training program. Dr Cremer earned a Bachelor’s degree in molecular biology from Princeton University and his medical degree from Harvard Medical School. Following completion of his internal medicine residency at Massachusetts General Hospital, he continued his postdoctoral training with a 3-year fellowship in cardiovascular medicine and a subsequent 2-year fellowship in advanced cardiovascular imaging, both at Cleveland Clinic. His clinical and research interests include auto-inflammatory cardiovascular disease.
